# Leptospira in river and soil in a highly endemic area of Ecuador

**DOI:** 10.1186/s12866-020-02069-y

**Published:** 2021-01-07

**Authors:** Erin Miller, Veronica Barragan, Jorge Chiriboga, Chad Weddell, Ligia Luna, Dulce J. Jiménez, John Aleman, Joseph R. Mihaljevic, Sonora Olivas, Jane Marks, Ricardo Izurieta, Nathan Nieto, Paul Keim, Gabriel Trueba, J. Gregory Caporaso, Talima Pearson

**Affiliations:** 1 The Pathogen and Microbiome Institute, Northern Arizona University, Flagstaff, AZ USA; 2 Department of Biological Sciences, Northern Arizona University, Flagstaff, AZ USA; 3 Universidad San Francisco de Quito, Colegio de Ciencias Biologicas y Ambientales, Instituto de Microbiologia, Quito, Ecuador; 4 College of Public Health, University of South Florida, Tampa, FL USA; 5 School of Informatics, Computing and Cyber Systems, Northern Arizona University, Flagstaff, AZ USA; 6 The Center for Ecosystem Science and Society, Northern Arizona University, Flagstaff, AZ USA

**Keywords:** Environmental detection of *Leptospira*, *Leptospira* in soil, *Leptospira* in water, Epidemiology of leptospirosis, Leptospirosis transmission

## Abstract

**Background:**

*Leptospira* are shed into the environment via urine of infected animals. Rivers are thought to be an important risk factor for transmission to humans, though much is unknown about the types of environment or characteristics that favor survival. To address this, we screened for *Leptospira* DNA in two rivers in rural Ecuador where Leptospirosis is endemic.

**Results:**

We collected 112 longitudinal samples and recorded pH, temperature, river depth, precipitation, and dissolved oxygen. We also performed a series of three experiments designed to provide insight into *Leptospira* presence in the soil. In the first soil experiment, we characterized prevalence and co-occurrence of *Leptospira* with other bacterial taxa in the soil at dispersed sites along the rivers (*n* = 64). In the second soil experiment, we collected 24 river samples and 48 soil samples at three points along eight transects to compare the likelihood of finding *Leptospira* in the river and on the shore at different distances from the river. In a third experiment, we tested whether *Leptospira* presence is associated with soil moisture by collecting 25 soil samples from two different sites.

In our river experiment, we found pathogenic *Leptospira* in only 4 (3.7%) of samples. In contrast, pathogenic *Leptospira* species were found in 22% of shore soil at dispersed sites, 16.7% of soil samples (compared to 4.2% of river samples) in the transects, and 40% of soil samples to test for associations with soil moisture.

**Conclusions:**

Our data are limited to two sites in a highly endemic area, but the scarcity of *Leptospira* DNA in the river is not consistent with the widespread contention of the importance of river water for leptospirosis transmission. While *Leptospira* may be shed directly into the river, onto the shores, or washed into the river from more remote sites, massive dilution and limited persistence in rivers may reduce the environmental load and therefore, the epidemiological significance of such sources. It is also possible that transmission may occur more frequently on shores where people are liable to be barefoot. Molecular studies that further explore the role of rivers and water bodies in the epidemiology of leptospirosis are needed.

## Background

With over one million cases estimated yearly, leptospirosis is among the world’s most common zoonoses with a mean case fatality ratio of 6.85% [1]. Pathogenic spirochaete species of the genus *Leptospira* have been detected in many geographic regions, but pose an especially prevalent threat in tropical and subtropical areas with poor sanitation infrastructure and increased exposure to infected wild, peri-domestic, and domestic animals [1–4].

The epidemiology of leptospirosis and ecology of *Leptospira* is particularly complex given the genetic diversity in the genus *Leptospira*. There are currently 64 species in the genus divided into two phylogenetic clades: 1) The “Saprophytic” clade contains species isolated from the environment that are not known to cause disease in humans and animals, and 2) the “Pathogenic” clade which includes species responsible for causing infections as well as species isolated from the environment with unknown virulence [5].

Survival of pathogenic *Leptospira* in the environment, after being shed in the urine of an infected animal, is critical for transmission to another host. Many human cases have been statistically attributed to river or lake exposure, suggesting high survival in larger bodies of water, however causal evidence is lacking and pathogenic leptospires have rarely been detected in these environments [6–21]. The soils bordering rivers may serve as a reservoir for these bacteria or otherwise reflect the presence of leptospires in rivers. Indeed, soil contamination has long been suspected as a possible source of *Leptospira* transmission and has received recent attention [22–25]. Given the perceived epidemiological importance of rivers, investigating the presence and survival of leptospires in river water and soil bordering rivers may better define where and how transmission in a riverine environment occurs.

Warm, humid climates and soil/water conditions are thought to be conducive to longer environmental survival times [3, 26]. Factors like high rainfall [27, 28], a neutral or slightly alkaline pH [6, 9, 11, 29], sediments in water [30, 31], low levels of human fecal contamination [30, 32], and high moisture in soil [9, 33] have been associated with the presence of *Leptospira*. Microbial communities in water and soil may also impact survival or indicate the presence of *Leptospira*. While different species of *Leptospira* may exhibit differential survival under different conditions, the extent of replication in the environment is largely unknown [34–36], despite the clear epidemiological importance of such knowledge. However, recent work with *L. interrogans* shows no replication in tested environmental microcosms, suggesting that the environment is only a temporary reservoir for this pathogen [22, 23]. In general, the roles and relative importance of environmental variables on *Leptospira* persistence are poorly understood, hindering the prediction of outbreaks.

We sought to investigate the presence of *Leptospira* in river water and soils from two highly endemic rural communities in Ecuador. In the towns of Calderón and Santa Ana, people and animals are dependent on nearby rivers for watering animals, drinking water, bathing, laundry, and transportation. Moreover, over the course of this study, leptospirosis cases were common in the community and we detected pathogenic *Leptospira* in both humans and animals [37]. We therefore hypothesized that we would readily detect pathogenic *Leptospira* in samples collected in these riverine environments.

## Methods

### Study site

This study is part of a broader research project on the prevalence of pathogenic *Leptospira* in humans and animal reservoir in the communities of Abdon Calderon (1°2′25″S, 80°22′15″W), and Santa Ana de Vuelta Larga (1°12′25″S 80°22′15″W) [37]. The two low-income, rural towns of Abdon Calderón (Site 1) and Santa Ana de Vuelta Larga (Site 2) are located near the coast in the Ecuadorian province of Manabí and approximately 20 km apart (S1 file). Prevalence of leptospirosis is high in this area with an average of ~ 448 human cases/year, higher than elsewhere in Ecuador (Barragan et al. unpublished). In these communities, multiple *Leptospira* species circulate among humans, cows, pigs, and rats [37]. Unlike urban communities where rats are thought to be the main source of leptospirosis, in these rural communities, the prevalence in rats is low (~ 3%) compared to livestock (~ 33%) and probably impacts the ecology and epidemiology of this disease [37]. Livestock and human interactions with the local rivers may be especially important as rivers are an established risk factor for leptospirosis [38].

The Chico River runs through Site 1 and the Portoviejo River runs through Site 2.

In order to explore the presence of pathogenic Leptospira in river water and soil samples, we used four different sampling strategies: 1) From each of these two rivers, we collected samples from sites upstream and downstream of the towns (described below in *River Experiment*). 2) From multiple sites along the shore of each river, we collected soil samples (described in *Soil Experiment #1*). 3) We intensively sampled soil from two sites, across a water-shore transect (*Soil Experiment #2*), and 4) intensively sampled soil at two locations to determine associations with soil moisture (*Soil Experiment #3*). All river collection sites are commonly used by the local population for swimming and watering their livestock (cattle, sheep and goats).

### River experiment – Leptospira prevalence and abiotic associations

**Fig. 1:**
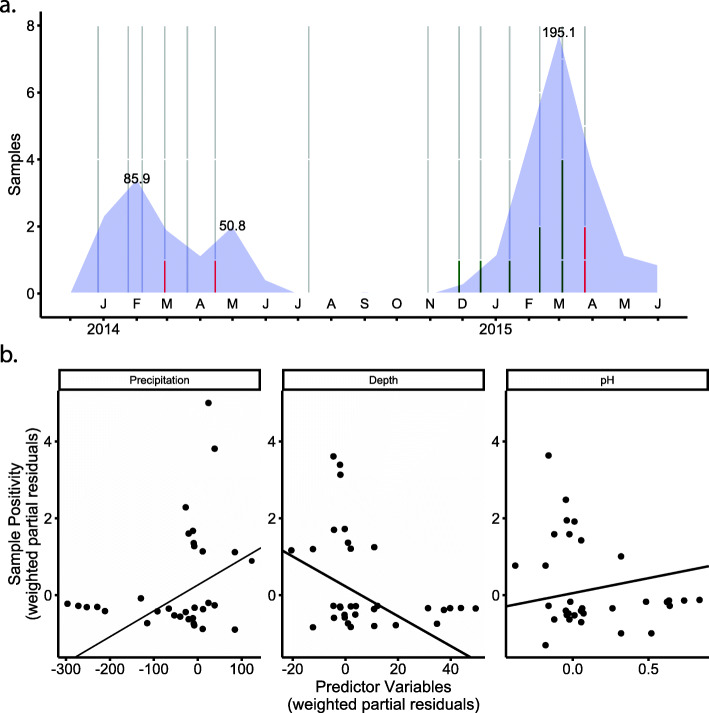
Positivity of *Leptospira* in river water. **a.** Eight river water samples (bars) were collected at each time point. Grey portion of bars represent no detection, green represents samples with saprophytic species and red represents samples where infectious species were detected. Monthly precipitation (mm) in Manabí province (blue) is overlaid on this timeline. **b.** Partial regression plots (also known as added-variable plots) for the partial effects of environmental covariates on *Leptospira* presence in river water. Note that sample positivity is represented by partial residuals. Precipitation, river depth, and pH all had statistically significant effects (Table 1). See S5 Table for the median values of dissolved oxygen, river depth, temperature, and pH

River water samples were collected between December of 2013 and March of 2015 (see Fig. 1 for sample collection timeline). When possible, sampling was performed in monthly intervals. For sites 1 and 2, the downstream sampling locations were ~ 1.6 km and 1.2 km (respectively) downriver from the upstream locations. At each time point, we collected 2 water samples (40 mL each) from the main current (not from near the shore) at both upstream and downstream sites in each river for a total of 14 time points and 112 river water samples (Fig. 1). All samples were immediately stored at 4 °C for transport and subsequently at − 20 °C until DNA extraction.

For each river, we used a GL500U-2-1 water sensor (Global Water, Gold River, CA) to log river depth on an hourly basis between February of 2014 and June of 2015. We also collected pH, dissolved oxygen (mg/L), and temperature (°C) of river water concurrent with sample collection, between July of 2014 and March of 2015.

### Soil experiment #1 - Leptospira prevalence and co-occurrence with other bacteria

We collected surface soil samples (no more than 3 cm in depth) from multiple sites along the shores of both rivers in July (*n* = 38) and August (*n* = 26) of 2014. Soil samples were collected on the shore 5 m from the river edge.

### Soil experiment #2 - Leptospira prevalence along water-shore transects

**Fig. 2:**
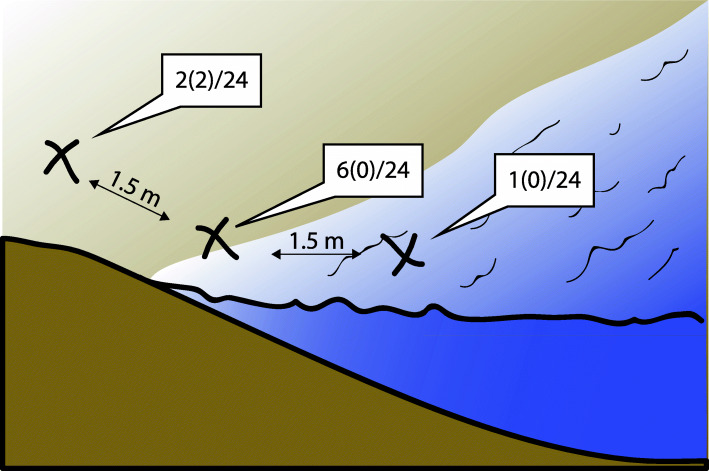
Results of water/shore transect sampling**.** In each of 4 transects in each of two rivers, we sampled 3 points in triplicate for a total of 72 samples. The number of samples in which infectious and saprophytic (in parentheses) *Leptospira* were detected are the numerator out of the total number of samples collected at each point in the transects (denominator) are shown. The exact location of the site was on the shore, immediately next to the river in an area that is commonly wet with waves from the river

In April and May of 2015, we collected samples along eight transects next to the Chico (4 transects) and Portoviejo Rivers (4 transects) (Fig. 2). Samples from each transect were collected at the same time point. Each transect had three sampling points: 1) water collected 1.5 m from the shore, 2) on the shore immediately next to the river, and 3) on the shore 1.5 m from the river. For the 2nd and 3rd positions in each transect, we collected the upper 5 mm of soil. For the 1st sampling positions, we collected 40 mL of river water as described above in our River Experiment. All samples were collected in triplicate for a total of 9 samples from each transect and 72 total samples. After collection, all samples were immediately stored at 4 °C for transportation and subsequently at − 20 °C until DNA extraction [39].

### Soil experiment #3 - Leptospira association with soil moisture content

In July 2018, we collected 25 soil samples at two sites within 1.5 m from the shore of the Chico River at the same location as the river sampling and two of the transect sites (Ecuadorian permits: MAE-DNB-CM-2018-0085 and MAE-CGZ4-DPAM-2018-1457-O). For each sample, approximately 150 g of soil was collected in a sterile ziplock bag and immediately stored at 4 °C for transportation: 100 g of soil was used for measuring soil water content (moisture). The weight of the sample was measured before and after drying with a Drier Box DHG-9030A at 105 °C for 24 h to determine the water content [40]. Samples DNA extraction was performed as described below, and tested using the SNP111 assay to detect infectious *Leptospira* [37].

### DNA extraction

DNA from individual soil samples was extracted using the PowerSoil® DNA Isolation Kit (MO BIO, Carlsbad, CA, USA), while DNA from river water samples was extracted using the PowerWater® DNA Isolation Kit (MO BIO, Carlsbad, CA, USA). DNA extraction from soil and water samples was performed in duplicate and triplicate, respectively. The manufacturer’s protocols were followed with the exception of the final elution into 2 mL tubes using 100 uL of Buffer AE (Qiagen, Valencia, CA, US), as opposed to Solutions C6 for soil and PW6 for water. This alteration was made due to the stabilizing effect of the EDTA content of Buffer AE’s composition (10 mM Tris-Cl; 0.5 mM EDTA, pH 9.0).

### Detection of infectious and saprophytic Leptospira

DNA extractions were then screened using the SNP111 TaqMan qPCR assay to detect members of the pathogenic and intermediate *Leptospira* clades [37], also referred to as P1 and P2 clades [41]. To detect the presence of member of the saprophytic *Leptospira* clade, also referred to as S1 file [41], we designed the Sapro assay, a single TaqMan® MGB probe assay (S1 file). All PCR assays were performed in quadruplicate.

### Inhibition testing

As a quality control for PCR, we sought to determine the extent to which our PCR was being inhibited, either by humic acids or excessive amounts of extracted DNA. 10% of samples from each sample type (e.g., water, soil) were randomly selected for inhibition testing. Each sample was divided into two aliquots: one was used as a control, and the other was spiked with *Leptospira* DNA such that it contained 1 μL of sample extraction template plus 1 μL of the positive control plasmid. Both aliquots were tested with the SNP111 Assay. The presence of inhibitors in a sample would be indicated by a diminished amplification of the 153 bp positive control target band. Samples did not show inhibition.

### 16S community sequencing

We investigated the microbial community structure for a subset of samples collected in *Soil Experiment #1* (*n* = 59 out of 64). Samples were indexed and prepared for sequencing according to the 16S rRNA Amplification Protocol of the Earth Microbiome Project [42]. All PCR products were cleaned with carboxylated magnetic beads, both before and after pooling. Upon observing an acceptable quantity and purity of the pooled samples, a quantitative PCR using Illumina DNA Standards was carried out to verify overall target DNA concentrations (Kapa Biosystems, Wilmington, MA). Samples were all sequenced on MiSeq Desktop Sequencers (Illumina Inc., San Diego, CA) using MiSeq Reagent Kits v2 at a read length of 2 × 150.

### Community analyses

Sequence data returned by each MiSeq run was processed using QIIME 22019.7 [43]. Sequence quality control and definition of amplicon sequence variants (ASVs) was performed with DADA2 [44] using the q2-dada2 QIIME 2 plugin. Sequences were processed with and without paired end read joining, and ultimately we chose to proceed with analysis of unpaired forward reads as read joining resulted in loss of approximately 50% of sequences. After definition of ASVs, sample replicates were collapsed by the site they were derived from by summing ASV counts across replicates. The Observed OTUs (OO), Faith’s Phylogenetic Diversity [45], unweighted UniFrac, and weighted UniFrac diversity metrics [46] were computed out using an even sampling (rarefaction) depth of 7164 sequences per sample. Taxonomy was assigned to ASV sequences using the q2-feature-classifier [47], Naïve Bayes classifier [47] and the Greengenes 13_8 reference database [48]. A phylogenetic tree was constructed using q2-fragment-insertion [49]. We next tested for differences in community composition (beta diversity) and community richness (alpha diversity), and for differentially abundant ASVs and genera across samples which had any positive *Leptospira* hits and those sites that didn’t have any positive *Leptospira* hits. Kruskal-Wallis was applied to test for differences in community richness, PERMANOVA for community composition, and ANCOM [50] for differentially abundant ASVs and genera.

### Statistical analyses

To test the effects of environmental covariates on the presence of *Leptospira* in river water, we conducted a logistic regression with a quasibinomial error distribution to account for under-dispersion in the data (dispersion parameter estimated as 0.75). We considered a sample to be positive if any of the two qPCR tests (SNP111 and Sapro assays) were positive, thus including members of the pathogenic, intermediate, and saprophytic *Leptospira* clades (see qPCR methods described above). We combined pathogenic, intermediate, and saprophytic *Leptospira* to increase the number of positive samples and because the shared evolutionary history of these *Leptospira* are likely to lead to shared ecological qualities. As such, environmental covariates for one type may shed light on others, independent of whether they cause disease in humans. We started with a full model that included the effects of total monthly precipitation, river depth, dissolved oxygen, pH, water temperature, and a fixed effect of river identity. All continuous covariates were centered by subtracting the mean before analysis. We then used step-wise model selection to determine the most parsimonious model. We dropped variables sequentially and conducted a likelihood ratio test to determine whether dropping variables was appropriate, using an alpha value of 0.05 as our drop threshold. The final model included total monthly precipitation, depth, pH, and river identity. All analyses were conducted in the open-source statistical programming software, *R* [51].

## Results

### Performance of the assay for detecting saprophytic *Leptospira*

The Sapro assay appears to be capable of detecting *Leptospira* species within the saprophytic clade. Extensive in silico analysis of 278 *Leptospira* 16S rRNA gene sequences that were publicly available at the time of assay design (July 2014) as well as 26 saprophytic species suggests that the Sapro assay is specific for species within the saprophytic clade (S1 file). The probe and forward primer bind perfectly to all 26 saprophytic species while the reverse primer contains a single mismatch with 2/26 species (S1 file). Also, laboratory tests showed that none of the infectious *Leptospira* species, or the 10 non-*Leptospira* species amplified with the assay (S1 file). However, *Leptonema illini* synthetic DNA was amplified by this assay, suggesting the possibility for false positive results. The Sapro assay exhibited a lowest limit of quantification (LoQ) and lowest limit of detection (LoD) to be ten 16S rRNA copies per microliter of extracted DNA in Qiagen AE elution buffer (S1 file). The range of linearity of the Sapro assay was 10^8 to10^1 16S rRNA genes.

#### River experiment – Leptospira prevalence and abiotic associations

We detected infectious *Leptospira* in four of 112 (3.6%) river water samples; however, only a very small amount of *Leptospira* DNA was present as all amplification curves appeared at very late cycles (average C_t_ of 35.7: range = 35–36.16). This average C_t_ corresponds to one 16S copy and is within the limit of detection of the SNP111 assay. We found evidence of saprophytic *Leptospira* in nine samples. As with our detection of infectious *Leptospira*, the Sapro assay suggests very small quantities of saprophytic DNA (average C_t_ of 35.96, corresponding to one 16S copy, C_t_ values ranged from 26.59–43.17). Unlike the SNP111 assay, C_t_ values above approximately 34 fall outside the limit of detection, indicating that saprophytic species are present at concentrations that are too low to produce reliable detection. While sampling took place from December of 2013 through March of 2015, *Leptospira* were nearly exclusively detected in wet months (Fig. 1).

We were more likely to detect *Leptospira* (saprophytic and infectious) when rivers were shallower and in months with higher total precipitation, and *Leptospira* was less likely to be found in water with high pH (Table 1, Fig. 1b). Although we found 7 positive samples in the Chico River (Site 1) and 6 positive samples in the Portoviejo River (Site 2), the model shows a higher likelihood of *Leptospira* presence in the Portoviejo River, after accounting for the other environmental covariates. This effect is likely due to the fact that, although *Leptospira* was more likely to be found when rivers were shallower, the Portoviejo River had on average higher depth compared to the Chico River (118.0 vs 90.0 cm, respectively). Therefore, to account for the positive samples found in the Portoviejo River at higher average depths, the model estimates an overall higher likelihood of detecting *Leptospira* in the Portoviejo River. Based on a separate logistic regression *a priori*, there was no difference in the proportion of positive samples detected upstream versus downstream (z = 1.439, *p* = 0.15). Our final model therefore accounted for 43.2% of the variability in the *Leptospira* river data, based on a pseudo-R^2^ using the Nagelkerke method [52, 53]. A summary of all four abiotic factors tested for each river (depth) or at each site (dissolved oxygen, temperature, and pH) are listed in S5 Table, which reports the median values of each one for those samples with *Leptospira* and for those without. Manabí Precipitation data were provided by the Instituto Nacional de Meteorologia e Hidrologia (INAMHI).

**Table 1:** Results of the logistic regression for the river samples, assessing the effects of precipitation, river depth, river pH, and river identity on the likelihood of detecting *Leptospira*. Significance of coefficients (slopes and intercepts) was evaluated with an alpha = 0.05

	***Coefficient***	***Std. Error***	***Odds Ratio***	***t-value***	***p-value***
*(Intercept)*	−3.321	0.824	0.036	−4.029	0.0002
*Precipitation*	0.020	0.007	1.021	2.788	0.007
*Depth*	−0.123	0.046	0.884	−2.667	0.010
*pH*	−5.289	2.204	0.005	−2.400	0.020
*River* *(Portoviejo* vs. *Chico)*	1.866	0.878	6.462	2.126	0.038

#### Soil experiment #1 - Leptospira prevalence and co-occurrence with other bacteria

Of the 64 dispersed soil samples collected in July and August of 2014, 14 were positive for infectious *Leptospira* and nine were positive for saprophytic *Leptospira*. In all cases, the detection was robust with an average C_t_ of 29.3 (range: 27–32) for infectious *Leptospira* and 27.4 (range: 24–32) for saprophytic *Leptospira* and seen in at least two of the four replicates for each sample.

Microbial community analysis of alpha diversity using Phylogenetic Diversity (PD) showed no difference in richness between samples that were positive versus negative for saprophytic *Leptospira* (Kruskal-Wallis H = 0.002, *p* = 0.959, q = 0.959); higher richness for soils that were positive for infectious Leptospira versus negative for infectious Leptospira (Kruskal-Wallis H = 8.19, *p* = 0.004, q = 0.014); and higher richness for samples that were positive for either infectious or saprophytic *Leptospira* versus negative for both (Kruskal-Wallis H = 6.19, *p* = 0.013, q = 0.026). Microbial community analysis of alpha diversity using Observed OTUs (OO) showed no difference in richness between samples that were positive versus negative for saprophytic *Leptospira* (Kruskal-Wallis H = 0.04, *p* = 0.838, q = 0.914); higher richness for soils that were positive for infectious Leptospira versus negative for infectious Leptospira (Kruskal-Wallis H = 11.58, *p* = 0.001, q = 0.012); and higher richness for samples that were positive for either infectious or saprophytic *Leptospira* versus negative for both (Kruskal-Wallis H = 7.50, *p* = 0.006, q = 0.014). PERMANOVA tests of unweighted UniFrac distances between samples illustrated minor differences in composition between samples that were positive versus negative for saprophytic *Leptospira* (pseudo-F = 1.34, *p* = 0.018, q = 0.031); minor differences in composition between samples that were positive versus negative for infectious *Leptospira* (pseudo-F = 1.44, *p* = 0.004, q = 0.014); and no difference in composition for samples that were positive for either infectious or saprophytic *Leptospira* versus negative for both (pseudo-F = 1.14, *p* = 0.127, q = 0.169). PERMANOVA tests of weighted UniFrac distances between samples illustrated minor differences in composition between samples that were positive versus negative for saprophytic *Leptospira* (pseudo-F = 2.54, *p* = 0.006, q = 0.014); no differences in composition between samples that were positive versus negative for infectious *Leptospira* (pseudo-F = 1.73, *p* = 0.064, q = 0.096); and no difference in composition for samples that were positive for either infectious or saprophytic *Leptospira* versus negative for both (pseudo-F = 0.90, *p* = 0.525, q = 0.630). While some of these results are suggestive of statistical significance, none are significant after Benjamini-Hochberg False Discovery Rate correction for the 12 results presented in this paragraph.

ANCOM analysis of the three sample groups (i.e., infectious, saprophytic, and infectious or saprophytic), identified taxa that were differentially abundant across the samples that were positive versus negative for each. An unidentified genus in the Acidobacterial order E29 (ANCOM W = 1034) and an unidentified genus in the NC10 (phylum) order MIZ17 (ANCOM W = 952) were present in higher abundance in infectious-*Leptospira*-positive samples than infectious-*Leptospira*-negative samples. The Cyanobacterial genus *Arthronema* (ANCOM W = 941) was present in higher abundance in the saprophytic-*Leptospira*-positive samples than saprophytic-*Leptospira*-negative samples. An unidentified genus in the Acidobacterial order E29 (W = 428) was also identified to be present in higher abundance in samples that were positive for either infectious or saprophytic *Leptospira* than in samples that were not negative for both. QIIME 2 artifacts and visualizations generated for microbiome analysis, and all QIIME 2 commands run for these analyses are available at https://github.com/caporaso-lab/ecuador-leptospira. The contained files can be viewed at https://view.qiime2.org.

#### Soil experiment #2 - Leptospira prevalence along water-shore transects

Infectious *Leptospira* were detected in 9 of 72 samples: 1 of 24 samples from the water and 8 of 48 from the shore (6 of 24 from the water-shore junction and 2 of 24 from higher on the shore). Saprophytic *Leptospira* was detected in only 2 of 72 samples, both from the shore (Fig. 2). We conducted a logistic regression to determine if any differences exist between the proportion of positive *Leptospira* samples discovered on the shore, at the water-shore junction, and in the river. For this analysis, we grouped infectious and saprophytic *Leptospira* types. We found that we were marginally more likely to find *Leptospira* at the water-shore junction compared to in the river (z = − 1.730, *p* = 0.083), and there was no difference between the proportion positive on the shore soil and at the water-shore junction (z = − 0.664, *p* = 0.507). Therefore, we are possibly more likely to find *Leptospira* in wet shore soil compared to in the river water itself.

#### Soil experiment #3 - Leptospira association with soil moisture content

High positivity of infectious *Leptospira* (40%) was detected in 25 shore soil samples collected within 1.5 m from the river. Positive samples had between 11.04 and 20.8% water content. Soils that tested positive for infectious *Leptospira* had a higher moisture content (M = 15.46, SD = 3.09) than samples that tested negative (M = 13.39, SD = 4.22), however, a logistic regression testing the effect of soil moisture on the presence of *Leptospira* did not show a significant effect (*z*-value = 1.186, *p* = 0.236; Fig. 1). This result is probably due to our small sample size and the narrow spread of observed moisture values.

## Discussion

### Leptospira in river water

Rivers have long been associated with the transmission of leptospirosis, however, our data suggest that the contact with soil, rather than river water itself, may be more likely to result in transmission of *Leptospira*. In 112 samples from two rivers in the highly endemic province of Manabí, Ecuador, DNA from both infectious and saprophytic *Leptospira* were scarcely observed. This is despite the centrality of the two rivers to communities with high rates of leptospirosis. The paucity of *Leptospira* DNA in rivers is indicative of the lack of viable *Leptospira*, without which, rivers cannot contribute to the epidemiology of leptospirosis. These findings are consistent with the limited success of other efforts to find evidence of *Leptospira* in river water, with almost all positive samples being found near the shore, in small streams, stagnant water, or at very low concentrations [6, 8–10, 24, 25, 30, 33, 35, 54–56] . A limitation of our river sampling work is that the volume collected was not guided by the ability to detect a target concentration based on the infectious dose of *Leptospira*. The infectious dose of *Leptospira* is not known [21], nor is the degree to which likelihood of infection may be modulated by exposure time. However our detection of ~ 0.0125 *Leptospira* per mL (based on results from the four positive samples) is likely to be many orders of magnitude below the infectious dose, even under prolonged exposure.

Given this scarcity of infectious *Leptospira* species in the river, we searched for abiotic associations with all *Leptospira* as the shared evolutionary history of infectious and saprophytic *Leptospira* are likely to have resulted in many similar ecological qualities. In our study, the observed range of water temperature and dissolved oxygen were not associated with sample positivity. The observed pH values (7.4–8.8) are within a range at which *Leptospira* have been found previously [6, 9, 11, 29], but slightly above values systematically tested for survival times [29]. Our results show a higher likelihood of finding *Leptospira* at the lower end of the observed range. *Leptospira* presence was also more likely in months where rainfall was high (see also [35]). This is consistent with an increase in leptospirosis cases following periods of high rainfall [27, 28, 57–59] which may be due to a general increase of viable *Leptospira* in the environment [59]. The increased likelihood of our detection of *Leptospira* when river depths were lower may be due to a decreased dilution effect of *Leptospira* as well as increased dependence on the river and tributaries by livestock with fewer alternative sources of surface water.

### Leptospira in shore soil

A much higher number of soil samples tested positive for infectious *Leptospira* (14 of 64 from *Soil Experiment #1*, 8 of 48 soil samples from *Soil Experiment #2*, and 10 of 25 soil samples from *Soil Experiment #3*) compared to river water samples (4 of 112 from our *River Experiment* and 1 of 24 of the river samples from *Soil Experiment #2*). Furthermore, the soil samples contained higher amounts of infectious *Leptospira* DNA. These results are consistent with a higher likelihood of finding infectious *Leptospira* in soils compared to canals and rivers [24]. These results are highly suggestive of the relative importance of rivers and soils for transmission and environmental cycling.

It is possible that microbial communities influence the survival and persistence of infectious and saprophytic *Leptospira*. However after False Discovery Rate correction, we found no significant difference in species richness and overall community composition between samples that were positive or negative for *Leptospira*. We did however identify 2 uncharacterized genera from the orders E29 and MIZ27 that were found in higher abundance in samples containing infectious *Leptospira*. The E29 genus was also at higher abundance in samples with saprophytic *Leptospira*. A third genus (Arthronema), was present in higher abundance in samples containing saprophytic *Leptospira*. Unfortunately little is known about the ecological roles of these genera, making it difficult to speculate about ecological conditions that may provide insights into the presence or absence of *Leptospira*.

Our intensive soil sampling from two sites (*Soil Experiment #3)* showed a correlation between infectious *Leptospira* positivity and soil moisture, however this was not statistically significant, likely due to our small sample size and lack of samples with low moisture content. Comparing the presence of *Leptospira* along three transects of the Chico and Portoviejo rivers showed that pathogenic species were more likely to be found in samples taken from the water-shore junction than in the water, though this effect was marginal. This observation is consistent with our results suggesting the importance of high moisture content for the survival of infectious *Leptospira*. Our findings are also consistent with previous work on the importance of soil, soil moisture, and small puddles for the presence of infectious *Leptospira* [9, 22, 24, 25, 60]. This, coupled with the likelihood of finding infectious *Leptospira* in other shore samples, as opposed to the river itself, adds to evidence pointing to river shores as a larger threat of infection in these endemic communities of Manabí, Ecuador.

### Overview

In this study, we aimed to explore the epidemiological importance of the riverine environment by testing for the presence of pathogenic *Leptospira* in the water column. Our results cast doubt upon the importance of river exposure in leptospirosis transmission. While many studies have demonstrated exposure to rivers as a risk factor [6–21], our findings lead us to conclude that the epidemiological role of rivers for *Leptospira* transmission may be limited compared to soils and river shores.

After being excreted via urine into the soil, direct contact with *Leptospira* contaminated soil may result in transmission. The likelihood of transmission will depend on the environmental persistence of sufficiently high concentrations of *Leptospira* needed to cause infection. If shed directly into a river or subsequently washed into one, *Leptospira* will be severely diluted, decreasing the likelihood of infection. Environmental persistence is critical, and rains may prime the soil to maximize *Leptospira* survival, thereby expanding the time window where contact can lead to transmission. This mechanism may explain our increased detection, as well as the higher number of clinical cases, associated with precipitation.

Our work here specifically addresses the role of the water column in rivers, but our conclusions may be generalizable to other parts of the river (near the shore or benthic area) as well as large bodies of water whereby entering *Lepospira* will be severely diluted. For example, strong epidemiological links to a reservoir led to subsequent investigations that only revealed molecular or microbiological evidence of *Leptospira* in the shore soils, rather than in the water itself [15]. Similar to our conclusions regarding rivers, we suspect that the epidemiological role of lakes and reservoirs is limited. We therefore hypothesize that water-associated behavior, which often entails walking barefoot on the shore, provides an avenue for contact with moist soil that may harbor high concentrations of *Leptospira* and result in transmission. Similarly, flooding is commonly associated with leptospirosis outbreaks as flood waters can contain and disperse high concentrations of *Leptospira* from overflowing sewers and flooded agricultural areas [61–63]. Importantly, the widespread tendency to remove shoes during floods may put high numbers of people at particular risk for infection.

There are a few notable limitations of this work and its implications. Firstly, river samples were collected from the water column and not the sediments. As such, we cannot assess the likelihood of *Leptospira* in benthic material. Secondly, there is still much to discover about the diversity of *Leptospira* and how *Leptospira* presence in the environment translates into human infections [64–66]. It is possible that a scarcity of the pathogen in rivers does not necessarily lead to a lower likelihood of transmission if, for instance, the presence of water makes skin more easily penetrable, or if highly concentrated biofilms occur. Thirdly, the infectious dose for any *Leptospira* species across any host is not known [21]. Fourth, this study incidentally captures the environmental circumstances at a time with abnormally low rainfall and less than average number of leptospirosis cases, possibly limiting how representative our results are of a typical year. Lastly, little is known about *Leptospira* survival in soils and the conditions in which it survives best. Despite these limitations, our work suggests that soil, rather than river exposure, may be more epidemiologically important. While these conclusions might be generalized across to other bodies of water, it is important to recognize the possibility, and associated epidemiological risk, for the accumulation of *Leptospira* in stagnant water sources [10, 11]. Additional work directed at testing the long-held assumption of the relevance of water exposure, and the importance of contact with moist soil should be prioritized as we work to more fully understand and control transmission.

## Supplementary Information

**Additional file 1 Fig. S1**. Map of study site.

**Additional file 2 S1 File**. Sapro assay design and validation.

**Additional file 3 Table S5.** Median values of abiotic river water measurements.

## Data Availability

The datasets used and/or analysed during the current study available at: 1) Sequences alignment of Leptospira spp. fragment amplified for Sapro assay http://purl.org/phylo/treebase/phylows/study/TB2:S27108?x-access-code=263709726a7b3481e9de706185eb102f&format=html. 2) Microbial community analysis https://www.dropbox.com/s/gb97xbv1ieija7z/barragan-et-al-sequence-data.zip?dl=0

## References

[1] Costa F, Hagan JE, Calcagno J, Kane M, Torgerson P, Martinez-Silveira MS, et al. Global Morbidity and Mortality of Leptospirosis: A Systematic Review. PLoS Negl Trop Dis. 2015;9(9):e0003898. 10.1371/journal.pntd.0003898. 10.1371/journal.pntd.0003898 PMC4574773 26379143

[2] McBride AJA, Athanazio DA, Reis MG, Ko AI (2005). Leptospirosis. Curr Opin Infect Dis.

[3] Levett PN (2001). Leptospirosis. Clin Microbiol Rev.

[4] Izurieta R, Galwankar S, Clem A (2008). Leptospirosis: The “mysterious” mimic. J Emerg Trauma Shock.

[5] Picardeau M. Leptospira and Leptospirosis. In: Methods in Molecular Biology: Humana Press Inc; 2020. p. 271–5. 10.1007/978-1-0716-0459-5_24. 10.1007/978-1-0716-0459-5_24 32632877

[6] Diesch SL, McCulloch WF (1966). Isolation of pathogenic leptospires from waters used for recreation. Public Health Rep.

[7] Wilkins E, Cope A, Waitkins S (1988). Rapids, rafts, and rats. Lancet.

[8] Thibeaux R, Geroult S, Benezech C, Chabaud S, Soupé-Gilbert M-E, Girault D (2017). Seeking the environmental source of Leptospirosis reveals durable bacterial viability in river soils. PLoS Negl Trop Dis.

[9] Saito M, Villanueva SYAM, Chakraborty A, Miyahara S, Segawa T, Asoh T (2013). Comparative analysis of Leptospira strains isolated from environmental soil and water in the Philippines and Japan. Appl Environ Microbiol.

[10] Sapian M, Khairi MT, How SH, Rajalingam R, Sahhir K, Norazah A, et al. Outbreak of Melioidosis and Leptospirosis Co-infection Following a Rescue Operation. Med J Malaysia. 2012;67 http://www.e-mjm.org/2012/v67n3/Melioidosis-and-Leptospirosis.pdf. Accessed 24 Jun 2019. 23082420

[11] Benacer D, Woh PY, Mohd Zain SN, Amran F, Thong KL (2013). Pathogenic and saprophytic Leptospira species in water and soils from selected urban sites in peninsular Malaysia. Microbes Environ.

[12] Stern EJ, Galloway R, Shadomy SV, Wannemuehler K, Atrubin D, Blackmore C (2010). Outbreak of Leptospirosis among Adventure Race Participants in Florida, 2005. Clin Infect Dis.

[13] Corwin A, Ryan A, BLoys W, Thomas R, Deniega B, Watts D (1990). A Waterborne Outbreak of Leptospirosis among United States Military Personnel in Okinawa, Japan. Int J Epidemiol.

[14] Katz AR, Manea SJ, Sasaki DM (1991). Leptospirosis on Kauai: investigation of a common source waterborne outbreak. Am J Public Health.

[15] Morgan J, Bornstein SL, Karpati AM, Bruce M, Bolin CA, Austin CC (2002). Outbreak of Leptospirosis among Triathlon Participants and Community Residents in Springfield, Illinois, 1998. Clin Infect Dis.

[16] Sejvar J, Bancroft E, Winthrop K, Bettinger J, Bajani M, Bragg S (2003). Leptospirosis in “Eco-Challenge” Athletes, Malaysian Borneo, 2000. Emerg Infect Dis.

[17] Boland M, Sayers G, Coleman T, Bergin C, Sheehan N, Creamer E (2004). A cluster of leptospirosis cases in canoeists following a competition on the River Liffey. Epidemiol Infect.

[18] Brockmann S, Piechotowski I, Bock-Hensley O, Winter C, Oehme R, Zimmermann S (2010). Outbreak of leptospirosis among triathlon participants in Germany, 2006. BMC Infect Dis.

[19] Hadad E, Pirogovsky A, Bartal C, Gilad J, Barnea A, Yitzhaki S (2006). An outbreak of leptospirosis among Israeli troops near the Jordan River. Am J Trop Med Hyg.

[20] Schreiber PW, Aceto L, Korach R, Marreros N, Ryser-Degiorgis M-P, Günthard HF (2015). Cluster of Leptospirosis Acquired Through River Surfing in Switzerland. Open Forum Infect Dis.

[21] Barragan V, Olivas S, Keim P, Pearson T. Critical knowledge gaps in our understanding of environmental cycling and transmission of Leptospira spp. Appl Environ Microbiol. 2017;83(19):e01190-17. 10.1128/AEM.01190-17. 10.1128/AEM.01190-17 PMC5601346 28754706

[22] Schneider AG, Casanovas-Massana A, Hacker KP, Wunder EA, Begon M, Reis MG (2018). Quantification of pathogenic Leptospira in the soils of a Brazilian urban slum. PLoS Negl Trop Dis.

[23] Casanovas-Massana A, Pedra GG, Wunder EA, Diggle PJ, Begon M, Ko AI (2018). Quantification of Leptospira interrogans Survival in Soil and Water Microcosms. Appl Environ Microbiol.

[24] Muñoz-Zanzi C, Mason MR, Encina C, Astroza A, Romero A (2014). Leptospira contamination in household and environmental water in rural communities in southern Chile. Int J Environ Res Public Health.

[25] Flores B, Escobar K, Muzquiz JL, Sheleby-Elías J, Mora B, Roque E, et al. Detection of pathogenic leptospires in water and soil in areas endemic to leptospirosis in Nicaragua. Trop Med Infect Dis. 2020;5. 10.3390/TROPICALMED5030149. 10.3390/tropicalmed5030149 PMC7559144 32962119

[26] Andre-Fontaine G, Aviat F, Thorin C (2015). Waterborne Leptospirosis: Survival and Preservation of the Virulence of Pathogenic Leptospira spp. in Fresh Water. Curr Microbiol.

[27] Ward MP (2002). Seasonality of canine leptospirosis in the United States and Canada and its association with rainfall. Prev Vet Med.

[28] Pappachan MJ, Sheela M, Aravindan KP (2004). Relation of rainfall pattern and epidemic leptospirosis in the Indian state of Kerala. J Epidemiol Community Health.

[29] Smith CE, Turner LH (1961). The effect of pH on the survival of leptospires in water. Bull World Health Organ.

[30] Viau EJ, Boehm AB (2011). Quantitative PCR-based detection of pathogenic Leptospira in Hawai’ian coastal streams. J Water Health.

[31] Chaturongkasumrit Y, Techaruvichit P, Takahashi H, Kimura B, Keeratipibul S (2013). Microbiological evaluation of water during the 2011 flood crisis in Thailand. Sci Total Environ.

[32] Chang SL, Buckingham M, Taylor MP (1948). Studies on Leptospira Icterohaemorrhagiae: IV. Survival in Water and Sewage: Destruction in Water by Halogen Compounds, Synthetic Detergents, and Heat. J Infect Dis.

[33] Henry RA, Johnson RC (1978). Distribution of the Genus Leptospira in Soil and Water.

[34] Barragan VA, Mejia ME, Trávez A, Zapata S, Hartskeerl RA, Haake DA (2011). Interactions of Leptospira with Environmental Bacteria from Surface Water. Curr Microbiol.

[35] Sato Y, Mizuyama M, Sato M, Minamoto T, Kimura R, Toma C (2019). Environmental DNA metabarcoding to detect pathogenic Leptospira and associated organisms in leptospirosis-endemic areas of Japan. Sci Rep.

[36] Vinod Kumar K, Lall C, Raj RV, Vedhagiri K, Vijayachari P (2015). Coexistence and survival of pathogenic leptospires by formation of biofilm with *Azospirillum*. FEMS Microbiol Ecol.

[37] Barragan V, Chiriboga J, Miller E, Olivas S, Birdsell D, Hepp C, et al. High Leptospira Diversity in Animals and Humans Complicates the Search for Common Reservoirs of Human Disease in Rural Ecuador. PLoS Negl Trop Dis. 2016;10(9):e0004990. 10.1371/journal.pntd.0004990. 10.1371/journal.pntd.0004990 PMC5021363 27622673

[38] Haake DA, Levett PN (2015). Leptospirosis in Humans.

[39] Weddell C (2015). Evaluation of Soil as a Risk Indicator for Human Leptospirosis in Coastal, Rural Ecuador.

[40] Lekshmi SU (2014). S, Singh DN, Shojaei Baghini M. A critical review of soil moisture measurement Measurement.

[41] Vincent AT, Schiettekatte O, Goarant C, Neela VK, Bernet E, Thibeaux R (2019). Revisiting the taxonomy and evolution of pathogenicity of the genus Leptospira through the prism of genomics. PLoS Negl Trop Dis.

[42] Caporaso JG, Lauber CL, Walters WA, Berg-Lyons D, Huntley J, Fierer N (2012). Ultra-high-throughput microbial community analysis on the Illumina HiSeq and MiSeq platforms. ISME J.

[43] Bolyen E, Rideout JR, Dillon MR, Bokulich NA, Abnet CC, Al-Ghalith GA, et al. Reproducible, interactive, scalable and extensible microbiome data science using QIIME 2. Nat Biotechnol. 2019. 10.1038/s41587-019-0209-9. 10.1038/s41587-019-0209-9 PMC7015180 31341288

[44] Callahan BJ, McMurdie PJ, Rosen MJ, Han AW, Johnson AJA, Holmes SP (2016). DADA2: High-resolution sample inference from Illumina amplicon data. Nat Methods.

[45] Faith DP (1992). Conservation evaluation and phylogenetic diversity. Biol Conserv.

[46] Lozupone CA, Hamady M, Kelley ST, Knight R (2007). Quantitative and qualitative beta diversity measures lead to different insights into factors that structure microbial communities. Appl Environ Microbiol.

[47] Bokulich NA, Kaehler BD, Rideout JR, Dillon M, Bolyen E, Knight R (2018). Optimizing taxonomic classification of marker-gene amplicon sequences with QIIME 2’s q2-feature-classifier plugin. Microbiome..

[48] McDonald D, Price MN, Goodrich J, Nawrocki EP, DeSantis TZ, Probst A (2012). An improved Greengenes taxonomy with explicit ranks for ecological and evolutionary analyses of bacteria and archaea. ISME J.

[49] Janssen S, McDonald D, Gonzalez A, Navas-Molina JA, Jiang L, Xu ZZ (2018). Phylogenetic Placement of Exact Amplicon Sequences Improves Associations with Clinical Information. mSystems.

[50] Mandal S, van Treuren W, White RA, Eggesbø M, Knight R, Peddada SD (2015). Analysis of composition of microbiomes: a novel method for studying microbial composition. Microb Ecol Health Dis.

[51] R Core Team (2018). R: A language and environment for statistical computing.

[52] Nakagawa S, Johnson PCD, Schielzeth H (2017). The coefficient of determination *R*^2^ and intra-class correlation coefficient from generalized linear mixed-effects models revisited and expanded. J R Soc Interface.

[53] Nagelkerke NJD (1991). A note on a general definition of the coefficient of determination. Biometrika..

[54] Anderson DC, Folland DS, Fox MD, Patton CM, Kaufmann AF (1978). Leptospirosis: A common-source outbreak due to leptospires of the grippotyphosa serogroup. Am J Epidemiol.

[55] Marinova-Petkova A, Guendel I, Strysko JP, Ekpo LL, Galloway R, Yoder J, et al. First Reported Human Cases of Leptospirosis in the United States Virgin Islands in the Aftermath of Hurricanes Irma and Maria, September–November 2017. Open Forum Infect Dis. 2019;6. 10.1093/ofid/ofz261. 10.1093/ofid/ofz261 PMC6602892 31289729

[56] Rawlins J, Portanova A, Zuckerman I, Loftis A, Ceccato P, Willingham AL (2014). Molecular detection of leptospiral DNA in environmental water on St. Kitts. Int J Environ Res Public Health.

[57] Gutiérrez JD, Martínez-Vega RA (2018). Spatiotemporal dynamics of human leptospirosis and its relationship with rainfall anomalies in Colombia. Trans R Soc Trop Med Hyg.

[58] Pawar SD, Kore M, Athalye A, Thombre PS (2018). Seasonality of leptospirosis and its association with rainfall and humidity in Ratnagiri, Maharashtra. Int J Health Allied Sci.

[59] Casanovas-Massana A, Costa F, Riediger IN, Cunha M, de Oliveira D, Mota DC (2018). Spatial and temporal dynamics of pathogenic Leptospira in surface waters from the urban slum environment. Water Res.

[60] Ganoza CA, Matthias MA, Collins-Richards D, Brouwer KC, Cunningham CB, Segura ER (2006). Determining risk for severe leptospirosis by molecular analysis of environmental surface waters for pathogenic Leptospira. PLoS Med.

[61] Trevejo RT, Rigau-Pérez JG, Ashford DA, McClure EM, Jarquín-González C, Amador JJ (1998). Epidemic Leptospirosis Associated with Pulmonary Hemorrhage—Nicaragua, 1995. J Infect Dis.

[62] Barcellos C, Sabroza PC (2001). The place behind the case: leptospirosis risks and associated environmental conditions in a flood-related outbreak in Rio de Janeiro. Cad Saúde Pública.

[63] Amilasan A-ST, Ujiie M, Suzuki M, Salva E, Belo MCP, Koizumi N (2012). Outbreak of leptospirosis after flood, the Philippines, 2009. Emerg Infect Dis.

[64] Guernier V, Goarant C, Benschop J, Lau CL (2018). A systematic review of human and animal leptospirosis in the Pacific Islands reveals pathogen and reservoir diversity. PLoS Negl Trop Dis.

[65] Thibeaux R, Iraola G, Ferrés I, Bierque E, Girault D, Soupé-Gilbert M-E (2018). Deciphering the unexplored Leptospira diversity from soils uncovers genomic evolution to virulence. Microb Genomics.

[66] Gorbea H, Garcia-Rivera EJ, Torres H, Lorenzi OD, Rivera A, Galloway RL (2018). Leptospirosis Cases Infected with Uncommon Serogroups, Puerto Rico, 2013-2015. Am J Trop Med Hyg.

