# Supplementary material for: Leptospira in river and soil in a highly endemic area of Ecuador
Source: BMC Microbiol. 2021 Jan 7;21:17. doi: 10.1186/s12866-020-02069-y (PMC7792295; doi:10.1186/s12866-020-02069-y)
Supplement: Supplementary file 1 — Additional file 1 Fig. S1. Map of study site. [file 12866_2020_2069_MOESM1_ESM.docx]

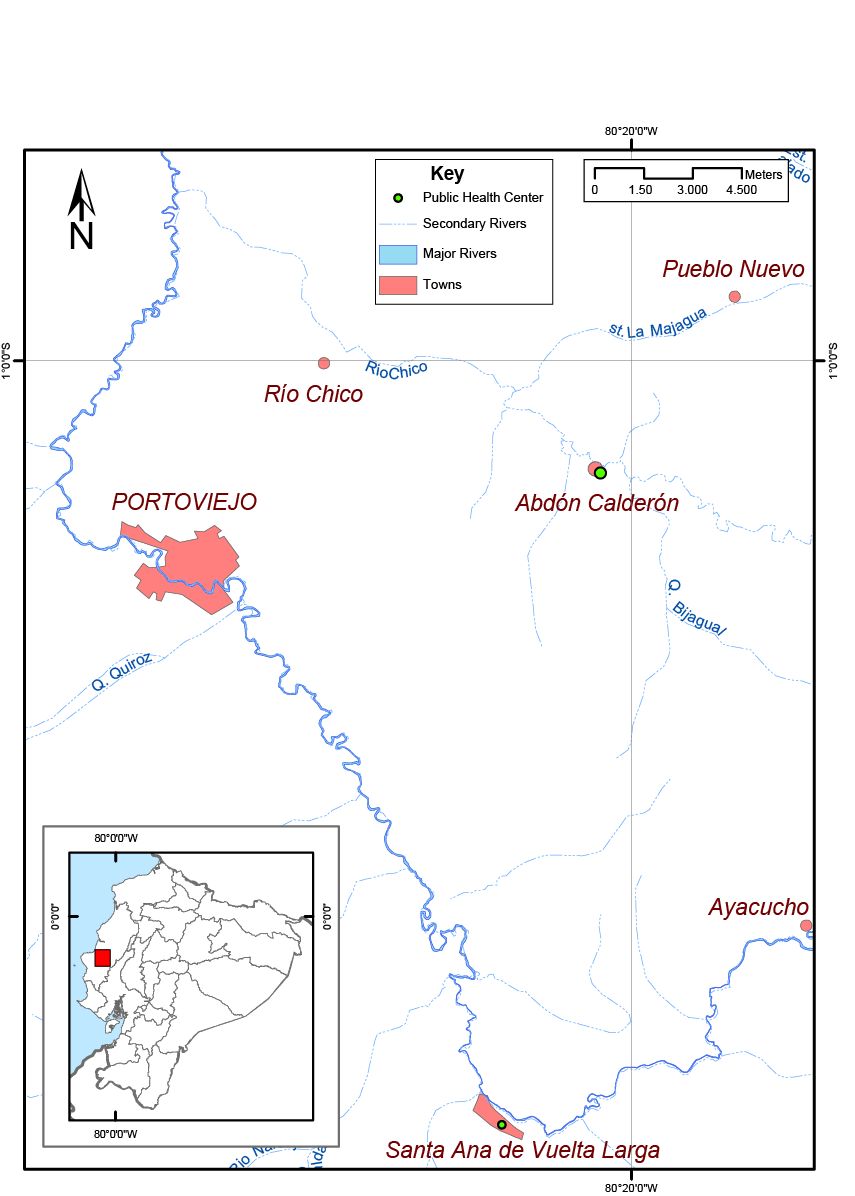


**S1 Figure**: Map of study site. The study site is located near the coast of Ecuador in the province of Manabi. Site 1 is the community of Abdon Calderon and Site 2 is Santa Ana de Vuelta Larga. This figure reproduced from Barragan et al. (2017).

Barragan V, Chiriboga J, Miller E, Olivas S, Birdsell D, Hepp C, Hornstra H, Schupp JM, Morales M, Gonzalez M, Reyes S, de la Cruz C, Keim P, Hartskeerl R, Trueba G, Pearson T. 2016. High Leptospira Diversity in Animals and Humans Complicates the Search for Common Reservoirs of Human Disease in Rural Ecuador. PLoS Neglected Tropical Diseases 10.
