# Supplementary material for: Leptospira in river and soil in a highly endemic area of Ecuador
Source: BMC Microbiol. 2021 Jan 7;21:17. doi: 10.1186/s12866-020-02069-y (PMC7792295; doi:10.1186/s12866-020-02069-y)
Supplement: Supplementary file 2 — Additional file 2 S1 File. Sapro assay design and validation. [file 12866_2020_2069_MOESM2_ESM.docx]

**Leptospira in river and soil in a highly endemic area of Ecuador.**

**Erin Miller^a,b^, Veronica Barragan^a,b,c^, Jorge Chiriboga^c^, Chad Weddell^d^, Ligia Luna^c^, Dulce J. Jiménez^b^, John Aleman^b^, Joseph R. Mihaljevic^e^, Sonora Olivas^a^, Jane Marks^b,f^, Ricardo Izurieta^d^, Nathan Nieto^b^, Paul Keim^a,b^, Gabriel Trueba^c^, J. Gregory Caporaso^a,b^, Talima Pearson^a,b^**

a. The Pathogen and Microbiome Institute, Northern Arizona University, Flagstaff, AZ

b. Department of Biological Sciences, Northern Arizona University, Flagstaff, AZ

c. Universidad San Francisco de Quito, Colegio de Ciencias Biologicas y Ambientales, Instituto de Microbiologia, Quito, Ecuador

d. College of Public Health, University of South Florida, Tampa, FL

e. School of Informatics, Computing and Cyber Systems, Northern Arizona University, Flagstaff, AZ

f. The Center for Ecosystem Science and Society, Northern Arizona University, Flagstaff, AZ

**Sapro assay design and validation**

Target sequence selection was performed as described by Barragan et al. 2017. In brief, we searched the 278 publicly available leptospira 16S rRNA gene sequences in the NCBI (http://www.ncbi.nlm.nih.gov) and JGI (https://img.jgi.doe.gov) databases for phylogenetically informative signatures among Leptospira spp. Sequences were aligned and single nucleotide polymorphisms (SNP) that provided discriminatory power among and within the “saprophytic” clade were identified. A target sequence of 153bp within the 16S rRNA gene was selected, corresponding to positions 3102729 to 3102577 and 1935913 to 1936065 in *Leptospira interrogans* serovar Lai str. 56609 (AE010300). Alignment was updated with newly discovered Leptospira species available in GeneBank.

In order to create positive controls and standardize quantification, a 330 bp copy of the 16S rRNA gene from *Leptospira biflexa* Patoc was synthesized as gBlocks gene fragments (IDT) and inserted inside the pCR 2.1 TOPO vector (Invitrogen Corp., Carlsbad, CA, USA). This fragment included the 153 bp fragment that provides identification and discrimination of “saprophytic” leptospira.

TaqMan® MGB probe and primers for the Sapro assay were designed using Primer Express® Software (Life Technologies) (S1 File Table 1). This assay was run on a 7900HT Fast Real-Time PCR System (Applied Biosystems) with SDS v2.4 software. A total reaction volume of 10 µl was prepared by using 1x TaqMan® Genotyping Master Mix (Applied Biosystems by Life Technologies, Foster City, CA, USA), 1µM of each primer, 300nM of Sapro probe (S1 File Table 1), and 1µl of DNA. Thermal cycling conditions were as follows: 50°C for 2 min., 95°C for 10 min., followed by 45 cycles of 95°C for 15 sec., 58°C for 90 sec. Positive controls and quantification standardization were performed using *Leptospira biflexa* Patoc as described by Barragan et al. 2016.

Assay metrics were determined by testing performance across several parameters: limit of quantification (LoQ) and detection (LoD), accuracy, precision, and specificity. The SNP signature used for the probe design was subjected to both *in silico* (BLAST analysis) and laboratory screening to determine specificity towards the saprophytic *Leptospira* clade (S1 File Table 2). For laboratory testing, the *Leptonema illini* 16S rRNA complete gene (chosen because it is the nearest neighbor of the genus Leptospira) was synthetized by gBlocks gene fragments (IDT) and inserted inside the pCR®2.1 TOPO® vector (Invitrogen Corp., Carlsbad, CA, USA) to keep it stable. DNAs from sixteen *Leptospira* species used in this study were supplied by the Royal Tropical Institute, Amsterdam, The Netherlands. DNA from non-*Leptospira* species, provided by Translational Genomics Research Institute (TGen) North (Flagstaff, AZ), were also tested to determine specificity of assays towards *Leptospira* species. These species included *Acinetobacter baumanii*, *Klebsiella pneumoniae*, *Staphylococcus epidermidis*, *Escherichia coli*, *Enterococcus faecalis*, *Enterobacter aerogenes*, *Moraxella catarrhalis*, *Streptococcus agalactiae*, *Neisseria meningitides*, and *Listeria monocytogenes* (S1 File Table 2).

Known quantities of control vectors were used to determine the lowest LoQ (S1 File Table 2) and LoD (S1 File Table 3). The lowest LoQ was defined when 4 of 4 replicates amplified with a cycle threshold (CT) of <0.3 standard deviation from the mean CT. The lower LoD was measured, after defining the lowest LoQ, as the lowest concentration of analyte that gave rise to signal (considering that negative controls gave no signal). Range of linearity of each assay was determined by 10-fold dilutions that resulted in a Ct separation of about 3.4.

| **Assay** | **Primers** | **Probe** | **Fragment size** | **Group detected** |
| --- | --- | --- | --- | --- |
| Sapro | F2: CGGCGCGTCTTAAACATG  R3B: TTACCCCACCAACTAGCTAATC | 6FAM-AAGCAGCAATGCGCTT | 207 bp | Saprophytic *Leptospira* spp. |

**S1 Table 1.** Real-time qPCR Sapro assay for detection of saprophytic *Leptospira* species.

**S1 Table 2.** Determination of Sapro assay specificity by comparison of in silico and Taqman real-time PCR against *Leptospira* non-*Leptospira* species.

**S1 Table 3.** Establishing the Lowest LoQ (limit of quantification) for Sapro assay. Lowest LoQ was determined as the lowest amount of 16S rRNA copies at which all 4 replicates amplified.

| **DNA** | **# of 16S rRNA gene copies** | **Replicates with amplification** |
| --- | --- | --- |
| *Leptospira biflexa* Patoc I | 10^5 | 4 of 4 |
|  | 10^4 | 4 of 4 |
|  | 10^3 | 4 of 4 |
|  | 10^2 | 4 of 4 |
|  | 10^1 | 4 of 4 |
|  | 10^0 | 3 of 4 |
|  | 10^-1 | 1 of 4 |
|  | 10^-2 | 0 of 4 |
| NTC | 0 | 0 of 10 |

**S1 Table 4.** Establishing the LoD (limit of detection) of Sapro assay with 11 replicates. Lowest LoD was determined as the lowest amount of 16S rRNA copies at which 8 of 11 replicates amplified

| **DNA** | **# of 16S copies** |  | **Replicates with signal** |
| --- | --- | --- | --- |
| *Leptospira biflexa* Patoc I | 10^1 |  | 11 of 11 |
|  | 10^0 |  | 5 of 11 |
|  | 10^-1 |  | 2 of 11 |
|  | 10^-2 |  | 0 of 11 |
| NTC | 0 |  | 0 of 11 |
