# Supplementary material for: Leptospira in river and soil in a highly endemic area of Ecuador
Source: BMC Microbiol. 2021 Jan 7;21:17. doi: 10.1186/s12866-020-02069-y (PMC7792295; doi:10.1186/s12866-020-02069-y)
Supplement: Supplementary file 3 — Additional file 3 Table S5. Median values of abiotic river water measurements. [file 12866_2020_2069_MOESM3_ESM.docx]

**S5 Table: Median values of abiotic river water measurements.** Measurements for dissolved oxygen, pH, temperature, and precipitation were collected between July 2014 and March 2015. Depth measurements were collected between February of 2014 and June of 2015.

| **Factor** | **Positive** | **Negative** |
| --- | --- | --- |
| **Depth (cm)** | 97.1 | 104.0 |
| **Dissolved Oxygen (mg/L)** | 4.08 | 4.53 |
| **pH** | 8.17 | 8.15 |
| **Temperature (°C)** | 28.6 | 28.5 |
| **Precipitation (mm)** | 119.0 | 53.0 |
